# PACS-2 attenuates diabetic kidney disease via the enhancement of mitochondria-associated endoplasmic reticulum membrane formation

**DOI:** 10.1038/s41419-021-04408-x

**Published:** 2021-11-26

**Authors:** Mei Xue, Ting Fang, Hongxi Sun, Ying Cheng, Ting Li, Chaofei Xu, Chao Tang, Xiaohuan Liu, Bei Sun, Liming Chen

**Affiliations:** 1 NHC Key Laboratory of Hormones and Development, Tianjin Key Laboratory of Metabolic Diseases, Chu Hsien-I Memorial Hospital & Tianjin Institute of Endocrinology, Tianjin Medical University, 300134 Tianjin, China; 2 Department of Endocrinology, Zhongnan Hospital of Wuhan University, 430071 Wuhan, China

**Keywords:** Type 1 diabetes, Type 2 diabetes, Diabetes complications

## Abstract

The altered homeostasis of mitochondria-associated endoplasmic reticulum (ER) membranes (MAM) was closely associated with the pathological process of nervous system diseases and insulin resistance. Here, the exact implication of phosphofurin acidic cluster sorting protein 2 (PCAS-2), an anchor protein in the MAM interface, in diabetic kidney disease was investigated. In the kidneys of type 1 and type 2 diabetes mice and HG-induced HK-2 cells, a notable disruption of ER-mitochondria interactions, accompanied by a decreased PACS-2 expression in all subcellular fractions. Furthermore, PACS-2 knockout mice with diabetes displayed accelerated development of proteinuria, deterioration of kidney function, and aggravated disruption of MAM area, ER stress, mitochondrial dysfunction, renal apoptosis, and fibrosis. However, overexpression of PACS-2 effectively protected diabetic kidneys and HG-treated HK-2 cells from renal tubular impairments. Importantly, experimental uncoupling of ER-mitochondria contacts reversed the protective effects of PACS-2 restoration on HK-2 cells under HG conditions. In summary, our data indicate a pivotal role of PACS-2 in the development of diabetic renal tubular injury via the stabilization of MAM.

## Introduction

Diabetes individuals are at increased risk for developing common microvascular complications, including diabetic kidney disease (DKD), and 30–50% of DKD patients will develop the end-stage renal disease [1]. DKD is a public health problem that will cause a significant social and economic burden, so new strategies should be urgently proposed to tackle this devastating situation [2]. The etiology of DKD is complicated and multifactorial, abnormal function of any kind of cells in the kidney may lead to diabetic kidney injury [3]. It has been well-documented that renal tubular epithelial cells play a critical role in the pathogenesis of DKD [4]. The damage of renal tubular epithelial cells is the main target and epicenter of renal fibrosis [5]. Diabetes-induced tubulointerstitial fibrosis was associated with proteinuria and deterioration of renal function [6].

Accumulating evidence suggests that dysfunction of cellular organelles contributes to the development of DKD [7, 8]. Mitochondria and endoplasmic reticulum (ER) are two important membrane structure organelles in cells, and the outer mitochondrial membrane and the ER membrane can be observed in close contact, which is called the mitochondrial-associated ER membrane (MAM) [9]. In physiological conditions, MAM is responsible for maintaining cellular homeostasis by the regulation of lipid metabolism, calcium signal, ER stress, mitochondrial function, apoptosis, and autophagy [10]. A large number of studies have demonstrated that MAM abnormalities may cause many neurodegenerative diseases [11, 12]. Additionally, MAM plays a crucial role in obesity- or diabetes-induced insulin resistance in liver and skeletal muscle [13–15].

Dozens of proteins including glucose-regulated protein 75 (Grp75), 1, 4, 5-trisphosphate receptor 1 (IP3R1), voltage-dependent anion channel 1 (VDAC1), and mitofusin 2 (Mfn2) are enriched in the MAM interface to maintain its structure and function [16]. Recently it was shown that MAM integrity is disrupted and DsbA-L expression is downregulated in the kidneys of DKD subjects, DsbA-L overexpression alleviates glucose-induced tubular injury by maintaining MAM integrity [17]. In addition, phosphofurin acidic cluster sorting protein 2 (PACS-2), another important mitochondrial-ER tether protein, affects the extent of mitochondria contact with ER. In obese animals, PACS-2 has a regulatory effect on mitochondrial oxidative capacity and hepatic insulin sensitivity [18]. A study has also found that PACS-2 is involved in oxidized low-density lipoprotein-induced endothelial cell apoptosis by regulating MAM contacts [19]. Disruption of MAM formation by PACS-2 knockdown hindered mitophagosome formation and potentiated vascular smooth muscle cell apoptosis [20]. However, it remains unclear how PACS-2 modulates the integrity of MAM and renal tubular function under diabetic conditions.

In the present study, we investigated the role of PACS-2 in MAM integrity and whether aberrant PACS-2 expression and MAM alterations could participate in the glucotoxicity-mediated renal tubular injury both in vivo and in vitro.

## Materials and methods

### Antibodies

The vendor and catalog number of the antibodies were listed in Supplementary Table 1.

### Animals

16-week-old male db/db mice and their non-diabetic db/m littermates were used as the type 2 diabetes models (*n* = 12 per group). For type 1 diabetes, 7-week-old male C57BL/6 mice were intraperitoneally injected with a single dose (150 mg/kg) of streptozocin (STZ) (*n* = 12 per group). One week after the injection, diabetes was successfully induced when the blood glucose level reached 16.7 mM for 3 consecutive days.

Male *Pacs-2* knockout (*Pacs-2*^−/−^) mice were obtained on C57BL/6 background. Wild-type (WT) mice and *Pacs-2*^−/−^ mice were randomly divided into non-diabetic and diabetic groups, respectively (*n* = 10 per group). Mice overexpressed PACS-2 were generated by the intravenous injection with adenovirus encoding for PACS-2 (Ad-PACS-2). Non-diabetic and diabetic mice were randomly injected Ad-PACS-2 or the empty virus vector (Ad-Con) (*n* = 10 per group).

When suffering from diabetes for 8 weeks, mice were killed after collecting the 24 h urine. Blood glucose was measured by Roche’s blood glucose meter. The kidney and liver function and blood lipid level were determined by an automatic biochemical analyzer (Roche, Germany). Urine biochemical analysis was performed using respective ELISA kits (Mlbio, China). All mice were acquired from the Model Animal Research Center of Nanjing University (Nanjing, China) and housed in the standard specific pathogen-free condition. All experiments were approved by the Ethics Committee of Tianjin Medical University (Tianjin, China).

### Cell culture

Human proximal tubular epithelial cells (HK-2) and human normal liver cells (HL-7702) were purchased from the Chinese Academy of Sciences Cell Bank (Shanghai, China) and cultured in DMEM medium supplemented with 10% fetal bovine serum. HK-2 cells were cultured either in 5.5 mM normal glucose (NG) or in 30 mM high glucose (HG) during 48 h, and 24.5 mM mannitol was added as the osmolality control. Cells were transfected with pCDNA3.1-foetal and adult testis expressed 1 (FATE1) plasmids, pCDNA3.1-PACS-2 plasmids, pCDNA3.1-PACS-2-Ires-FATE1 plasmids, or the plasmids vector using Lipofectamine 2000 reagent. In addition, HK-2 and HL-7702 cells were treated with palmitate to detect the effect of high lipid on PACS-2 expression in HK-2 and HL-7702 cells.

### Real-time PCR analysis

Total RNA was extracted, and cDNA was synthesized by reverse transcription. RT-PCR was performed using SYBR Green dye with specific primers on the CFX96 Manager system (Bio-Rad, USA). Primer sequences were listed in Supplementary Table 2. The mRNA level of target gene expression was corrected to β-actin.

### Western blot analysis

Protein lysates were prepared in RIPA buffer, and protein expression was analyzed by SDS-PAGE. After being incubated with primary antibodies and the secondary antibodies, the membranes were visualized using the enhanced chemiluminescence system. The intensities of immunoblot bands were quantified using ImageJ software.

### Renal histological analysis

Kidney sections were stained with HE, picrosirus red, and PAS according to the manufacturer’s protocols. For immunohistochemical analysis, kidney sections were deparaffinized, rehydrated, repaired, blocked, and incubated with the primary antibodies. After being incubated with the secondary antibodies, the sections were visualized by diaminobenzidine and finally counterstained with hematoxylin.

### Immunofluorescence analysis

HK-2 cells were fixed with 4% paraformaldehyde, permeabilized with 0.1% Triton X-100, blocked with 1% BSA, and incubated with the primary and secondary antibodies. After be counterstaining with DAPI, images were viewed by a fluorescence microscope (Olympus, Japan).

### Subcellular fractionation

See Supplementary Materials and Methods for details.

### Transmission electron microscopy

See Supplementary Materials and Methods for details.

### Mitochondrial membrane potential measurement

The mitochondrial membrane potential (MMP) of HK-2 cells was assessed using JC-1 dye (Solarbio, China). HK-2 cells were stained with the JC-1 working solution at 37 °C for 20 min and washed with diluted JC-1 staining buffer. Immediately, fluorescence images were captured with a fluorescence microscope (Olympus, Japan), and JC-1 red/green ratio was quantified by Image-Pro Plus 6.0 software.

### Mitochondrial DNA copy number measurement

See Supplementary Materials and Methods for details.

### In situ proximity ligation assay

The proximity ligations of VDAC1 and IP3R1 or GRP75 and IP3R1 were assayed by in situ proximity ligation assay (PLA; Sigma, USA). Briefly, paraffin-embedded kidney sections or coverslips planted with HK-2 cells were permeabilized, blocked, and incubated with primary antibodies. Then the kidney sections or cells were incubated with the PLA probes, ligation solution, and amplification solution. After staining the nuclei, images were captured by a light microscope (Olympus, Japan) or a confocal microscope (Carl Zeiss, Germany). In situ PLA dots per cell were counted using ImageJ software.

### TUNEL assay

TUNEL assay kit (Beyotime Biotechnology, China) was used to detect renal apoptosis. Briefly, kidney sections were dewaxed and then incubated with proteinase K. Each section was stained with a TUNEL reaction mixture, including 5 μl TdT and 45 μl fluorescein-labeled dUTP solution. Finally, cell nuclei were counterstained with DAPI.

### Flow cytometry

Annexin V-FITC/PI Apoptosis Detection Kit (BestBio, China) was used to assess the apoptosis of HK-2 cells. Cells were collected and resuspended in Binding Buffer. After incubating with Annexin V-FITC for 15 min and PI for 5 min at 4 °C in the dark, the samples were analyzed by a flow cytometer (ThermoFisher, America).

### Statistical analysis

Statistical analysis was conducted using GraphPad Prism 7.0 software. Data were expressed as the mean ± SD. Statistical significance was defined as *P* value < 0.05. Statistical comparisons between two groups were performed by unpaired Student’s *t* test. Comparisons among multiple groups were performed by one-way analysis of variance (ANOVA) followed by a post hoc Turkey’s test.

## Results

### MAM area was reduced and PACS-2 was downregulated in the kidneys of diabetic mice

**Fig. 1:**
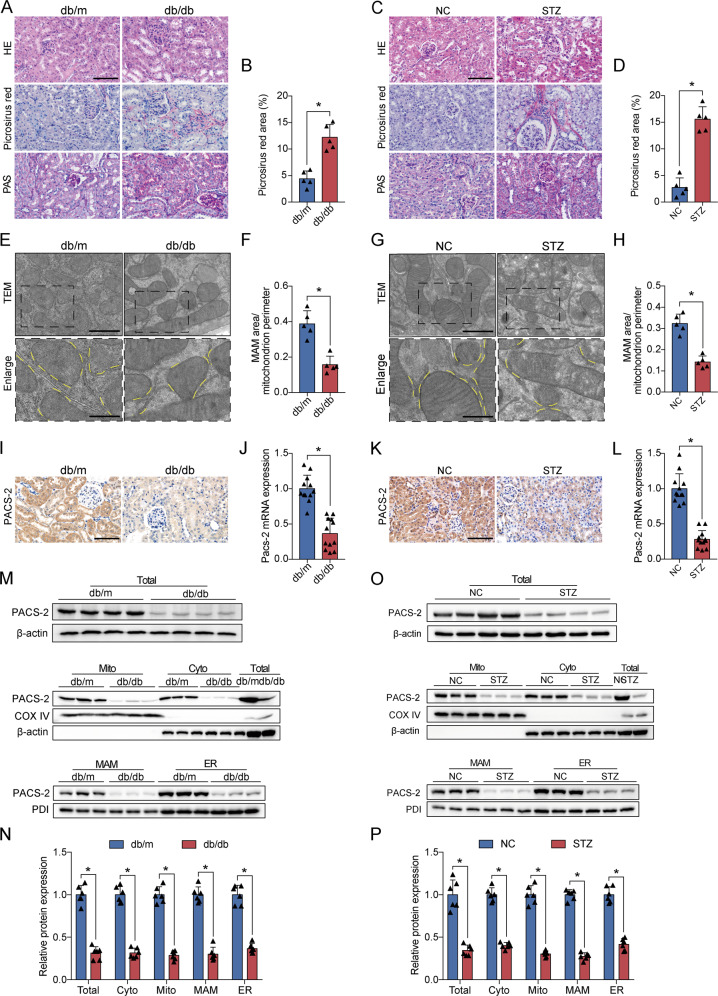
Diabetes induced disruption of the MAM interface and decreased PACS-2 expression in the kidneys. **A**, **C** Representative images of HE, Picrosirius red, and PAS staining of kidney sections. Scale bar = 100 μm. **B**, **D** Quantitative analysis of Picrosirius red staining in **A** and **C**. *n* = 5. **E**, **G** Representative TEM images showing the MAM area in the kidneys isolated from non-diabetic and diabetic mice. Yellow lines indicate the MAM interface. Scale bars = 1 μm, inset scale bar = 500 nm. **F**, **H** Quantitative analysis of MAM area normalized to mitochondrion perimeter in **E** and **G**. *n* = 5. **I**, **K** Immunohistochemical staining of PACS-2 in the kidney sections. Scale bar = 100 μm. **J**, **L** RT-PCR analysis of *Pacs-2* mRNA levels in the kidneys. *n* = 12. **M**, **O** Western blot bands of PACS-2 proteins in total lysates and subcellular fractions isolated from mice kidneys. **N**, **P** Quantitative analysis of PACS-2 protein levels in **M** and **O**. *n* = 6–8. All data are expressed as mean ± SD. *P < 0.05.

Compared with the non-diabetic mice, the diabetic mice had significant glomerular enlargement, mesangial matrix expansion, tubular hypertrophy and dilation, and tubular basement membrane disruption. Picrosirius red staining also revealed a notable increase in tubulointerstitial fibrosis in diabetic mice (Fig. 1A–D). The electron microscope images in proximal renal tubular cells showed that the proportion of ER in close contact with mitochondria relative to the mitochondrial perimeter was significantly lower in both diabetes models than in their controls (Fig. 1E–H).

PACS-2, as a MAM linking protein, was mainly distributed in renal tubules, and total PACS-2 mRNA and protein levels were significantly downregulated in the diabetic kidneys (Fig. 1I–P). However, the expression of PACS-2 in the liver of diabetic mice was increased (Supplementary Fig. 1A–B). To quantify the expression of PACS-2 in the MAM area, we isolate protein components of subcellular fractions prepared from kidneys. As shown in Supplementary Fig. 2, GRP75, as a mitochondrial stress protein, was observed mainly in the mitochondria and also was found in the cytoplasm and MAM. VDAC1 was enriched in mitochondrial fractions and IP3R1 was preferentially found in the ER. VDAC1 and IP3R1 can also be present in the MAM fraction. PDI, as ER marker and MAM marker, were equally expressed in MAM and ER. Mfn2 was present in all fractions. COX IV, as a mitochondrial marker, was only distributed in mitochondrial fractions. β-actin, as a cytosolic marker, was absent in mitochondria, MAM, and ER. PACS-2 was detected in isolated cytoplasm, mitochondria, MAM, and ER, and the renal PACS-2 expression levels were decreased in these subcellular fractions derived from diabetic animals compared with the non-diabetic mice (Fig. 1M–P).

### PACS-2 deficiency in diabetic mice decreased renal MAM integrity and exacerbated DKD

**Fig. 2:**
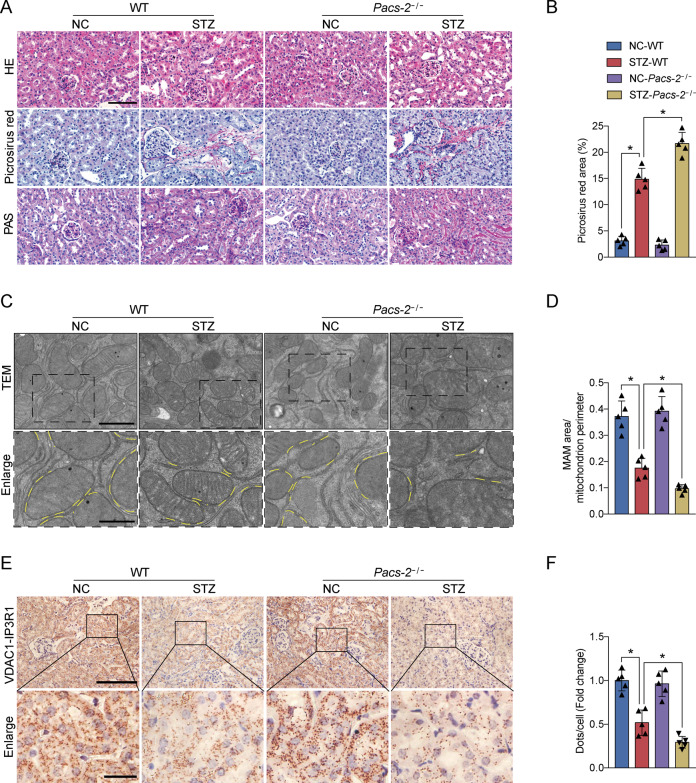
PACS-2 ablation aggregated histopathological changes and reduced MAM formation in diabetic kidneys. **A** Representative images of HE, Picrosirius red, and PAS staining of kidney sections. Scale bar = 100 μm. **B** Quantitative analysis of Picrosirius red staining in **A**. *n* = 5. **C** Representative TEM images showing the MAM area in the kidneys. Yellow lines indicate the MAM interface. Scale bars = 1 μm, inset scale bar = 500 nm. **D** Quantitative analysis of MAM area normalized to mitochondrion perimeter in **C**. *n* = 5. **E** Representative in situ PLA images showing the VDAC1/IP3R1 interactions in the kidneys. Scale bars = 100 μm, inset scale bar = 20 μm. **F** Quantitative analysis of in situ PLA dots in **E**. *n* = 5. All data are expressed as mean ± SD. *P < 0.05.

As displayed in Supplementary Fig. 3A–B, PACS-2 knockout mice did not express PACS-2 protein. As expected, STZ-induced diabetic mice showed an increase in blood glucose level and a decrease in body weight compared to control mice (Supplementary Table 3). Kidney weight to body weight ratio (KW/BW), urinary albumin excretion (UAE), urinary albumin to creatinine ratio (ACR), *N*-acetyl-β-d-glucosaminidase (NAG), serum creatinine (Scr), and blood urea nitrogen (BUN) were significantly increased in diabetic mice in comparison with non-diabetic mice (Supplementary Table 3). Interestingly, diabetic *Pacs-2*^*−/*^^−^ mice showed an obvious increase in KW/BW, UAE, ACR, NAG, Scr, and BUN levels relative to diabetic mice (Supplementary Table 3). There were no differences in the levels of body weight, blood glucose, alanine aminotransferase (ALT), aspartate aminotransferase (AST), triglyceride (TG), and total cholesterol (TC) between the diabetic and diabetic *Pacs-2*^*−/−*^ mice (Supplementary Table 3). The diabetes-associated tubular changes and tubulointerstitial fibrosis were more severe and the ER-mitochondria interaction sites were less in the diabetic *Pacs-2*^*−/−*^ group than in the diabetic group (Fig. 2A–D). In addition, in situ PLA was performed to analyze the mutual combination of VDAC1 and IP3R1 for visualizing the mitochondrial and ER contact. Consistently, compared with the diabetic group, the least VDAC1/IP3R1 complexes were found in the diabetic *Pacs-2*^*−/−*^ group (Fig. 2E, F).

**Fig. 3:**
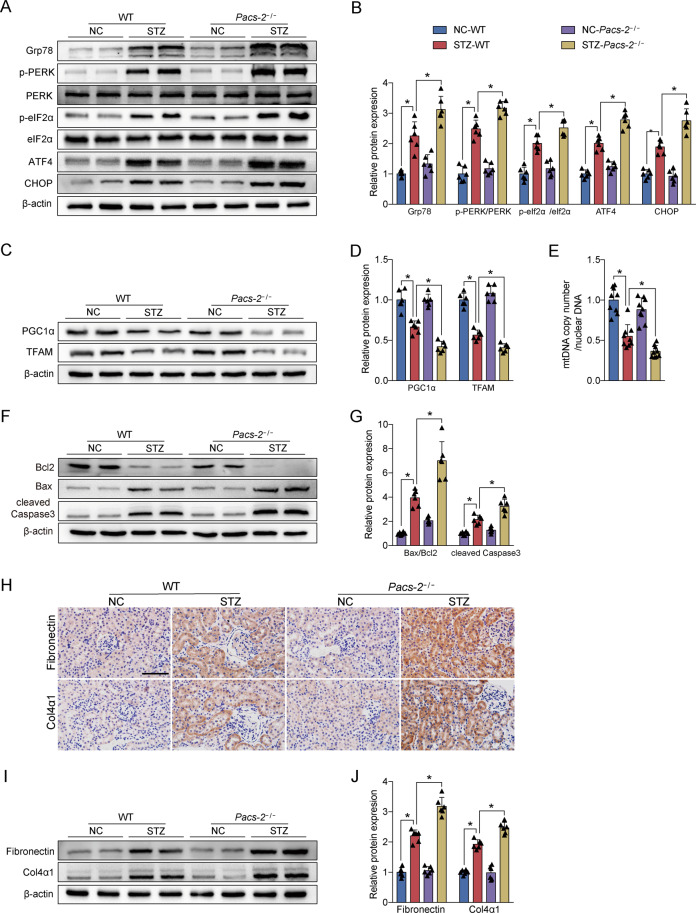
Diabetes-associated ER stress, mitochondrial dysfunction, apoptosis, and fibrosis were exacerbated in PACS-2 knockout kidneys. **A** Western blot bands of Grp78, p-PERK, PERK, p-eIF2α, eIF2α, ATF4, and CHOP in kidney lysates. **B** Quantitative analysis of indicated protein levels in **A**. *n* = 6. **C** Western blot bands of TFAM and PGC1α in kidney lysates. **D** Quantitative analysis of indicated protein levels in **C**. *n* = 6. **E** Measurement of mtDNA copy number normalized to the nuclear gene. *n* = 9. **F** Western blot bands of Bax, Bcl2, and cleaved Caspase3 in kidney lysates. **G** Quantitative analysis of indicated protein levels in **F**. *n* = 6. **H** Immunohistochemical staining of Fibronectin and Col4α1 in the kidney sections. Scale bar = 100 μm. **I** Western blot bands of Fibronectin and Col4α1 in kidney lysates. **J** Quantitative analysis of indicated protein levels in (**I**). *n* = 6. All data are expressed as mean ± SD. *P < 0.05.

The expression levels of the ER stress protein including Grp78, p-PERK/PERK, p-eIF2α/eIF2α, ATF4, and CHOP were notably increased in the diabetic group compared with the control group, whereas they were further increased in the diabetic *Pacs-2*^*−/−*^ group (Fig. 3A, B). Diabetic mice also exhibited an impairment in mitochondrial function accompanied by decreased levels of TFAM, PGC1α, and mitochondrial DNA (mtDNA) copy number, while the mitochondrial function was worsened in diabetic mice with PACS-2 deletion (Fig. 3C–E). Furthermore, in comparison with non-diabetic mice, diabetic mice showed marked renal apoptosis and fibrosis as indicated by a substantially elevated proportion of apoptotic cells and upregulated expression of Bax/Bcl2, cleaved Caspase3, Fibronectin, and Col4α1. Interestingly, renal apoptosis and fibrosis levels were further enhanced in diabetic *Pacs-2*^*−/−*^ mice (Fig. 3F–J and Supplementary Fig. 4A).

### PACS-2 overexpression in diabetic mice increased renal MAM integrity and rescued DKD

**Fig. 4:**
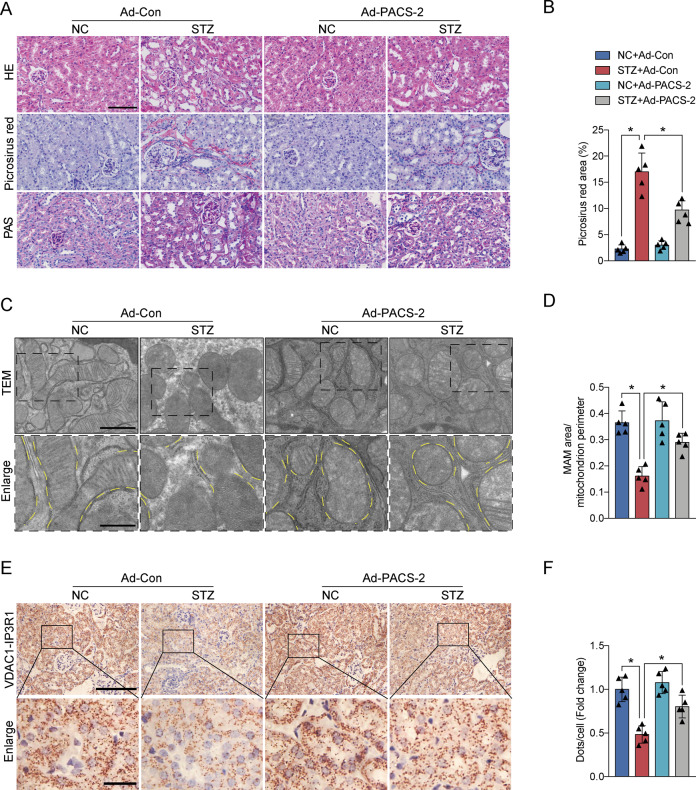
Renal histopathological changes were improved and MAM formation was enhanced after PACS-2 overexpression. **A** Representative images of HE, Picrosirius red, and PAS staining of kidney sections. Scale bar = 100 μm. **B** Quantitative analysis of Picrosirius red staining in **A**. *n* = 5. **C** Representative TEM images showing the MAM area in the kidneys. Yellow lines indicate the MAM interface. Scale bars = 1 μm, inset scale bar = 500 nm. **D** Quantitative analysis of MAM area normalized to mitochondrion perimeter in **C**. *n* = 5. **E** Representative in situ PLA images showing the VDAC1/IP3R1 interactions in the kidneys. Scale bars = 100 μm, inset scale bar = 20 μm. **F** Quantitative analysis of in situ PLA dots in **E**. *n* = 5. All data are expressed as mean ± SD. *P < 0.05.

As shown in Supplementary Fig. 3C–D, PACS-2 expression in the tubular cells of STZ-induced mice was significantly recovered following injection with Ad-PACS-2. The diabetic mice administered with Ad-PACS-2 displayed a decrease in KW/BW, UAE, ACR, NAG, Scr, and BUN levels compared to diabetic mice (Supplementary Table 4). The levels of body weight, blood glucose, liver function, and blood lipid were not significantly altered among all groups (Supplementary Table 4). Additionally, the diabetic mice treated with Ad-PACS-2 exhibited ease in diabetes-induced renal histopathological changes (Fig. 4A, B). Transmission electron microscopy (TEM) and in situ PLA analyses indicated that a remarkable increase was found in the MAM area in diabetic kidney overexpressed PACS-2 compared with diabetic mice (Fig. 4C–F).

**Fig. 5:**
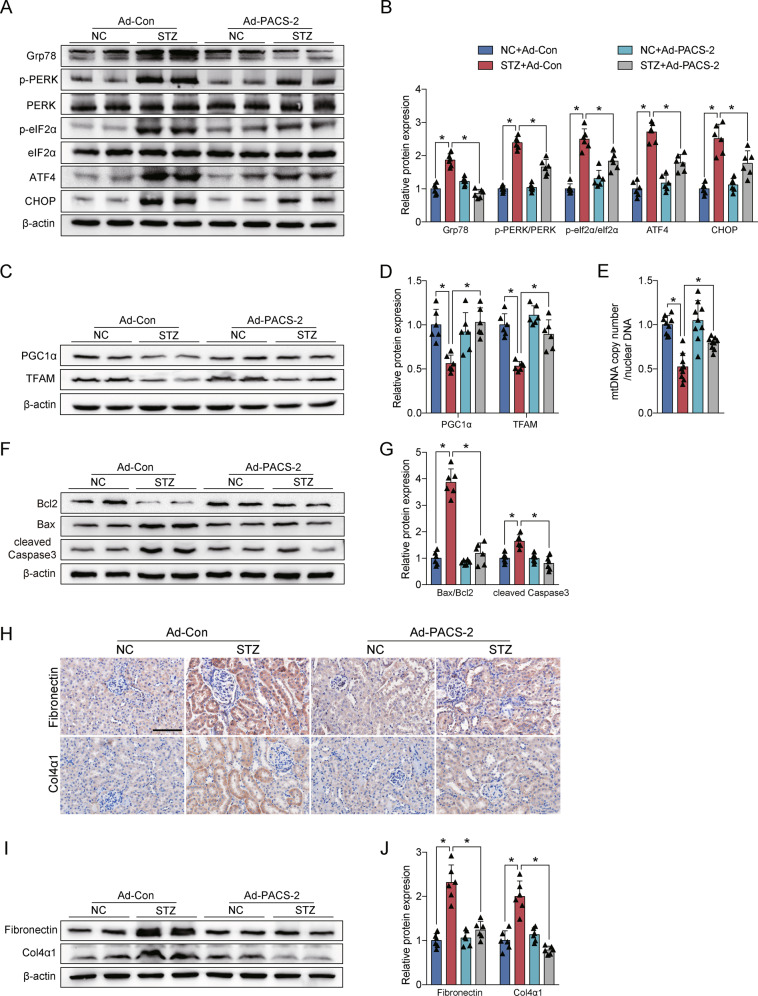
PACS-2 restoration attenuated ER stress, mitochondrial dysfunction, apoptosis, and fibrosis in diabetic kidneys. **A** Western blot bands of Grp78, p-PERK, PERK, p-eIF2α, eIF2α, ATF4, and CHOP in kidney lysates. **B** Quantitative analysis of indicated protein levels in **A**. *n* = 6. **C** Western blot bands of TFAM and PGC1α in kidney lysates. **D** Quantitative analysis of indicated protein levels in **C**. *n* = 6. **E** Measurement of mtDNA copy number normalized to nuclear gene. *n* = 9. **F** Western blot bands of Bax, Bcl2, and cleaved Caspase3 in kidney lysates. **G** Quantitative analysis of indicated protein levels in **F**. *n* = 6. **H** Immunohistochemical staining of Fibronectin and Col4α1 in the kidney sections. Scale bar = 100 μm. **I** Western blot bands of Fibronectin and Col4α1 in kidney lysates. **J** Quantitative analysis of indicated protein levels in **I**. *n* = 6. All data are expressed as mean ± SD. *P < 0.05.

The protein expression levels of Grp78, p-PERK/PERK, p-eIF2α/ eIF2α, ATF4, and CHOP were markedly decreased in the STZ + Ad-PACS-2 group relative to the STZ group (Fig. 5A, B). Meanwhile, the overexpression of PACS-2 in diabetic mice enhanced renal levels of TFAM, PGC1α, and mtDNA copy number (Fig. 5C–E). In addition, the proportion of apoptotic cells and expression of Bax/Bcl2, cleaved Caspase3, Fibronectin, and Col4α1 were significantly decreased in the STZ + Ad-PACS-2 group compared with the STZ group (Fig. 5F–J and Supplementary Fig. 4B).

### MAM integrity and PACS-2 expression were decreased under HG conditions in vitro

**Fig. 6:**
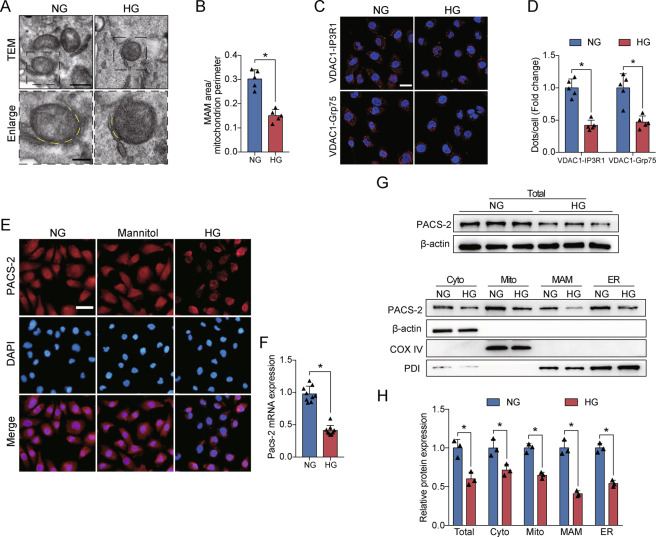
HG altered MAM integrity and PACS-2 expression in vitro. **A** Representative TEM images showing the MAM area in HK-2 cells. Yellow lines indicate the MAM interface. Scale bars = 500 nm, inset scale bar = 200 nm. **B** Quantitative analysis of MAM area normalized to mitochondrion perimeter in **A**. *n* = 5. **C** Representative in situ PLA images showing the VDAC1/IP3R1 or VDAC1/Grp75 complexes in HK-2 cells. Scale bars = 50 μm. **D** Quantitative analysis of in situ PLA dots in **D**. *n* = 5. **E** Immunofluorescence staining of PACS-2 in HK-2 cells. Scale bar = 50 μm. **F** RT-PCR analysis of *Pacs-2* mRNA levels in HK-2 cells. *n* = 9. **G** Western blot bands of PACS-2 proteins in total lysates and subcellular fractions isolated from HK-2 cells. **H** Quantitative analysis of PACS-2 protein levels in **G**. *n* = 3. All data are expressed as mean ± SD. *P < 0.05.

The population of MAM surface area was decreased in HG-treated HK-2 cells (Fig. 6A, B). Similar to TEM results, HG stimulation led to a significant reduction of VDAC1/Grp75 or VDAC1/IP3R1 interactions compared with the NG group (Fig. 6C, D). PACS-2 mRNA and protein levels were markedly diminished in HK-2 cells after exposure to HG, but mannitol treatment did not cause the decrease of PACS-2 fluorescence intensity (Fig. 6E–H). We then verified that the levels of PACS-2 were also significantly reduced in isolated cytoplasm, mitochondria, MAM, and ER fractions from HK-2 cells in response to HG stimulation (Fig. 6G, H). However, palmitate caused an increase in the expression of PACS-2 in both HK-2 cells and HL-7702 cells (Supplementary Fig. 1C–F).

### Effects of PACS-2 overexpression and MAM integrity on HK-2 cells injury

**Fig. 7:**
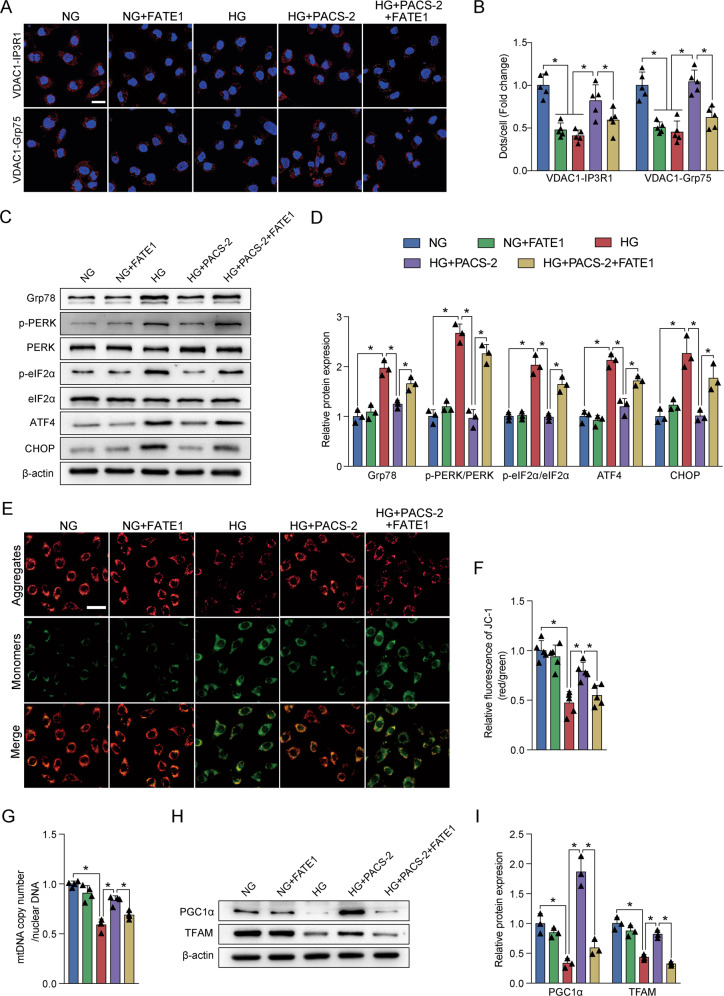
Experimental uncoupling ER-mitochondria interactions impaired the effects of PACS-2 overexpression on MAM homeostasis, HG-induced ER stress, and mitochondrial dysfunction. **A** Representative in situ PLA images showing the VDAC1/IP3R1 or VDAC1/Grp75 complexes in HK-2 cells. Scale bars = 50 μm. **B** Quantitative analysis of in situ PLA dots in **A**. *n* = 5. **C** Western blot bands of Grp78, p-PERK, PERK, p-eIF2α, eIF2α, ATF4, and CHOP in HK-2 cells. **D** Quantitative analysis of indicated protein levels in **C**. *n* = 3. **E** Mitochondrial membrane potential in HK-2 cells measured by JC-1 dye. Scale bar = 50 μm. **F** Quantitative analysis of JC-1 red fluorescence to JC-1 green ratio in **E**. *n* = 5. **G** Measurement of mtDNA copy number normalized to nuclear gene in HK-2 cells. *n* = 4. **H** Western blot bands of TFAM and PGC1α in HK-2 cells. **I** Quantitative analysis of indicated protein levels in **H**. *n* = 3. All data are expressed as mean ± SD. *P < 0.05.

FATE1, as an uncoupler of MAM, was not expressed in HK-2 cells. However, overexpressing PACS-2 and FATE1, separately or simultaneously, can successfully increase their protein levels relative to cells transfected with an empty vector (Supplementary Fig. 5). Compared with the control group, HG treatment or FATE1 transfection in HK-2 cells significantly reduced the VDAC1/Grp75 or VDAC1/IP3R1 complexes at the MAM interface. Importantly, overexpression of PACS-2 relieved the effect of HG on MAM surface area (Fig. 7A, B). Moreover, co-overexpression of FATE1 counteracted the restoration of ER-mitochondrial contacts by PACS-2 overexpression in the context of HG (Fig. 7A, B).

**Fig. 8:**
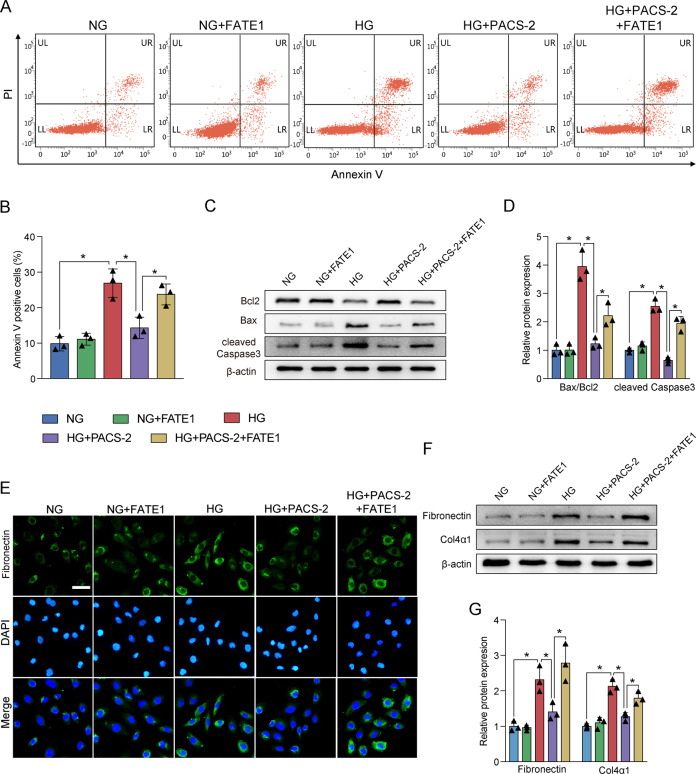
MAM integrity was required for the suppression of HG-induced apoptosis and fibrosis. **A** Representative images of flow cytometry in HK-2 cells. **B** Quantitative analysis of percent of apoptotic cells in **A**. *n* = 3. **C** Western blot bands of Bax, Bcl2, and cleaved Caspase3 in HK-2 cells. **D** Quantitative analysis of indicated protein levels in **A**. *n* = 3. **E** Immunofluorescence staining of Fibronectin in HK-2 cells. Scale bar = 50 μm. **F** Western blot bands of Fibronectin and Col4α1 in HK-2 cells. **G** Quantitative analysis of indicated protein levels in **F**. *n* = 3. All data are expressed as mean ± SD. *P < 0.05.

In vitro, HG also increased the expression of Grp78, p-PERK/PERK, p-eIF2α/ eIF2α, ATF4, and CHOP, and reduced the expression of TFAM and PGC1α as well as depleted the MMP and mtDNA copy number. However, PACS-2 overexpression significantly inhibited the increased levels of ER stress markers and restored the reduced levels of mitochondrial function markers (Fig. 7C–I). Additionally, flow cytometry analysis showed that HG led to an increase in HK-2 cell apoptosis (Fig. 8A, B). The dramatic upregulation of Bax/Bcl2 and cleaved Caspase3 levels were also induced by HG stimulation, which was accompanied by a discernible increase in expression of Fibronectin and Col4α1. Similarly, PACS-2 overexpression prevented HG-induced apoptosis and fibrosis (Fig. 8C–G). Surprisingly, co-overexpression of FATE1 moderately mitigated the protective effects of PACS-2 overexpression (Figs. 7C–I and 8A–G).

## Discussion

Inter-organelle miscommunication has recently emerged as critical regulator of multiple diseases. Studies have shown the importance of crosstalk between ER and mitochondria for neurodegenerative diseases [21]. Altered MAM interaction finely regulated the process of autophagy [22, 23]. The expression profile of cerebral MAM proteins extracted from type 2 diabetes animals significantly differed from those of non-diabetes [24]. Moreover, recent evidence indicates that MAM can be a control center for insulin action and glucose metabolism [25, 26]. In our study, MAM contact was significantly decreased in type 1 and type 2 diabetic kidneys and HG-treated HK-2 cells, which was in agreement with the previous study [17].

IP3R1-Grp75-VDAC1, the multi-protein complex that resided in the MAM interface, transfers Ca^2+^ flux from ER to mitochondria [27]. Mitochondrial Ca^2+^ concentration is essential for maintaining its function and cell survival, and transient fluctuations can destroy the balance of cell metabolism due to the narrow physiological range [28, 29]. Disruption of inter-organelle Ca^2+^ transfer was related to ER stress and mitochondrial dysfunction in hepatic insulin resistance [30]. Glucotoxicity can notably induce perturbations of mitochondrial Ca^2+^ signaling and subsequent organelles stress in β cells and neuronal cells via the IP3R1-VDAC1 interaction [31, 32]. In vitro, our data detected remarkably reductions in IP3R1-VDAC1 and Grp75-VDAC1 interactions after treatment with HG, thereby implicating that glucotoxicity caused the descending delivery of Ca^2+^ to mitochondria in HK-2 cells. HG-triggered MAM formation and mitochondrial Ca^2+^ turbulence were also involved in the pathology of diabetic cardiomyopathy [33]. Similarly, we observed that impairments of the MAM formation and IP3R1-Grp75-VDAC1 complexes were accompanied by ER stress, compromised mitochondrial function, and apoptosis both in vivo and in vitro. Aberrant of mitochondrial function is in the determination of cell death and triggers kidney tubular injury [34]. Furthermore, activation of PERK/eIF2α/ATF4/CHOP pathway induces pro-apoptotic Bax expression and suppresses anti-apoptotic Bcl-2 expression, ultimately resulting in caspase activation and apoptosis [35]. As a result, these adverse factors collaboratively contribute to the progression of DKD and eventually to renal fibrosis.

Ablation of anchoring protein in MAM affects cell biology by changing the distance between the ER and mitochondria [36]. PACS-2 was previously identified by its role in linking the ER-mitochondria axis to ER homeostasis, mitochondrial function, and apoptosis [37]. Recently, literature has reported the critical role of PACS-2 in metabolic disorders such as obesity and insulin resistance [18, 38]. We found an insufficient PACS-2 expression in all subcellular fractions in the kidney of type 1 and type 2 diabetes mice and HG-stimulated HK-2 cells, which is similar to a recent report that PACS-2 expression is decreased in different stages of DKD patients [39]. However, the expression of PACS-2 exhibits organ specificity and has different responses to high glucose or lipid. Overall, despite the conflicting results, the importance of PACS-2-related MAM homeostasis is unanimously recognized by various researchers, and whether PACS-2 has protective or harmful properties depends on the type of cell and stimulus [40]. Furthermore, PACS-2 deficiency accelerated the progression of diabetic kidney function and disruption of MAM interfaces, whereas adenoviral PACS-2 overexpression alleviated diabetes- and HG- associated ER stress, mitochondrial dysfunction, and renal tubular apoptosis and fibrosis. Therefore, we proposed that PACS-2 may appear as a major regulator of diabetic renal tubular injury via the enhancement of MAM formation. In addition to MAM, PACS-2 is also expressed in the cytoplasm, mitochondria, and ER and thus PACS-2 may regulate cell physiological functions in other ways. In TRAIL-induced apoptosis, endosomal PACS-2 recruits Bim and Bax to lysosomes resulting in the activation of executioner caspases [41]. On the other hand, the phosphorylation state of PACS-2 at the Ser437 site was considered as a molecular switch of TRAIL-induced apoptosis [42]. PACS-2 also modulates SIRT1-mediated deacetylation of p53 to induce p21-dependent cell cycle arrest following DNA damage [43].

FATE1, a cancer-testis antigen, can regulate the distance and Ca^2+^ flow between ER and mitochondria [44]. The present study showed that FATE1 transfection significantly increased the distance between the ER and mitochondria, and the protective effects of PACS-2 overexpression on apoptosis and fibrosis under HG were counteracted by FATE1-mediated organelle uncoupling, which is consistent with the results of other studies [13, 17]. Thus, adequate PACS-2 expression is a pivotal component of proper ER and mitochondrial function by the maintenance of ER-mitochondrial interaction in the diabetic kidneys. As previously reported in the kidneys of diabetic patients and animals, MAM integrity was negatively correlated with renal apoptosis and proteinuria [17]. Schneeberger et al. found that loss of ER-mitochondria contacts in hypothalamic neurons promotes ER stress [45]. Increasing MAM integrity may prevent ER stress and skeletal muscle insulin resistance [13], but another similar study reported that ER-mitochondria contact also could promote mitochondrial dysfunction and ER stress during obesity [15]. In the hepatic insulin resistance model, the MAM interface was also a crucial involvement in insulin signaling via inhibiting ER stress and mitochondrial dysfunction [14, 46]. Interestingly, acute HG exposure triggers mitochondrial bioenergetics and ATP synthesis of β cells, while chronic HG exposure may induce ER stress and mitochondrial dysfunction because of the depletion of ER Ca^2+^ stocks [31]. Therefore, the role of MAM homeostasis in the pathogenesis of disease may vary from different stress sources and animal models. Too loose or too tight ER-mitochondrial coupling may affect the transmission of calcium signals and cause dangerous stimuli to cells [47]. Based on the above findings, we believe that the structure of MAM is dynamic and MAM integrity needs to be maintained within a certain range to exert normal cellular activities.

However, further studies are needed to elucidate the mechanism of glucotoxicity-induced decreased expression of PACS-2 under diabetic conditions. Taken together, our results indicate that PACS-2 is a key molecule required for maintaining MAM homeostasis in diabetic renal tubular injury, and may represent a potential therapeutic target for DKD patients.

## Supplementary information

Supplemental files

## Data Availability

The datasets generated during the study are available from the corresponding author on reasonable request.
