# Supplementary material for: PACS-2 attenuates diabetic kidney disease via the enhancement of mitochondria-associated endoplasmic reticulum membrane formation
Source: Cell Death Dis. 2021 Nov 26;12(12):1107. doi: 10.1038/s41419-021-04408-x (PMC8626491; doi:10.1038/s41419-021-04408-x)
Supplement: Supplementary file 1 — Supplemental files [file 41419_2021_4408_MOESM1_ESM.pdf]

PACS-2 attenuates diabetic kidney disease via the enhancement of mitochondria-associated endoplasmic reticulum membrane formation

Mei Xue[1,2], Ting Fang[1], Hongxi Sun[1], Ying Cheng[1], Ting Li[1], Chaofei Xu[1], Chao Tang[1], Xiaohuan Liu[1], Bei Sun[1*], Liming Chen[1*].

[1] NHC Key Laboratory of Hormones and Development, Tianjin Key Laboratory of Metabolic Diseases, Chu Hsien-I Memorial Hospital & Tianjin Institute of Endocrinology, Tianjin Medical University, Tianjin 300134, China.

[2] Department of Endocrinology, Zhongnan Hospital of Wuhan University, Wuhan 430071, China.

*** Correspondence:**

Liming Chen, M.D., Ph.D. E-mail: xfx22081@vip.163.com. Tel.: +86-13920948158.

Bei Sun, M.D., Ph.D. E-mail: sun_peipei220@hotmail.com. Tel.: +86-18702209602.

Address: No.6 North Huanrui Rd, Beichen District, Tianjin, China.

Running title: PACS-2-induced MAM formation attenuates DKD.

Supplementary Materials and Methods

Subcellular fractionation

Briefly, kidney tissues or HK-2 cells were homogenized after washing. The homogenate was centrifuged twice at 740g for 5 min to discard the pellet containing unbroken cells and nuclei. The remaining supernatant was collected and centrifuged 3 times at 10000g for 10 min, and then the supernatant containing ER fraction and the pellet containing crude mitochondria fraction were separated. The ER fraction was gathered following centrifugation of the supernatant at 100000g for 60 min. The crude mitochondria pellet was suspended in mitochondrial resuspension buffer (MRB), layered in 30% Percoll medium, and further centrifuged at 95000g for 30 min. The pure mitochondria fraction, at the bottom of the tube, was diluted with MRB buffer and centrifuged twice at 6300 g for 10 min to collect the pellet. The MAM fraction, as an intermediate layer, was diluted with MRB buffer and purified by centrifugation at 100000g for 60 min. All pellets of the fractions were resuspended MRB buffer. MRB Buffer: 250mM Mannitol, 5mM HEPES, 0.5mM EGTA, pH 7.4. Percoll medium: 225mM Mannitol, 25mM HEPES, 1mM EGTA, 30% volume of Percoll (Sigma, USA), pH 7.4.

Transmission electron microscopy (TEM)

1 mm^3 fresh kidney tissues or HK-2 cells from 10 cm dish was harvested and immediately fixed with 2.5% glutaraldehyde, and postfixed 1% osmium tetroxide. After washing, samples were dehydrated, embedded, and polymerized. Ultrathin

sections were cut and stained with uranyl acetate and lead citrate. The structure of MAM was finally visualized under a transmission electron microscope. The mitochondria membrane in contact with ER within 30 nm was considered as MAM area. The measurement of mitochondrial perimeter and MAM area was used with ImageJ software.

Mitochondrial DNA (mtDNA) copy number measurement

Total DNA was extracted using genomic DNA extraction kit (Solarbio, China) according to the manufacturer's recommendations. Relative mtDNA copy number was measured by RT-PCR. Nicotinamide adenine dinucleotide dehydrogenase subunit 1 (ND1) was the mitochondrial gene and cytoglobin (CYGB) represents the reference single-copy nuclear gene. Primer sequences of ND1 and CYGB were listed in Table S2. The mtDNA copy number was normalized to the nuclear DNA.

Supplementary Tables

Supplementary Table 1. The source of the antibodies used in this study.

Antibodies	Vendor	Catalog number
PACS-2	Proteintech	19508-1-AP
Grp75	Proteintech	14887-1-AP
PDI	Proteintech	11245-1-AP
COX IV	Proteintech	11242-1-AP
Grp78	Proteintech	11587-1-AP
ATF4	Proteintech	10835-1-AP
TFAM	Proteintech	22586-1-AP
Bcl2	Proteintech	26593-1-AP
Fibronectin	Proteintech	66042-1-Ig
FATE1	Proteintech	23809-1-AP
IP3R1	Invitrogen	PA1-901
PERK	Invitrogen	PA5-99447
p-PERK (Thr982)	Invitrogen	PA5-102853
VDAC1	Abcam	ab14734
PGC1α	Abcam	ab191838
collagen IVα1 (Col4α1)	Abcam	ab227616
β-actin	Abcam	ab6276
β-tubulin	Abcam	ab6046
Mfn2	CST	9482
eIF2α	CST	9722
p-eIF2α (Ser 51)	CST	9721
CHOP	CST	2895
Bax	CST	2772
cleaved Caspase3	CST	9664

Supplementary Table 2. Primer sequences used for RT-PCR.

Gene	Primer sequences (5'-3')
Hsa PACS-2 Forward	GAGGAGCCAGCTTCAGGTTAG
Hsa PACS-2 Reverse	TCCGAGAGACAATGGTGCTG
Hsa β-actin Forward	CTCACCATGGATGATGATATCGC
Hsa β-actin Reverse	CACATAGGAATCCTTCTGACCCA
Mus PACS-2 Forward	AGATTGGGAGCATCCACAGC
Mus PACS-2 Reverse	GAGGGCCACCGTGTCAGAG
Mus β-actin Forward	CCTCTATGCCAACACAGTGC
Mus β-actin Reverse	ACATCTGCTGGAAGGTGGAC
Hsa mt-ND1 Forward	CTTCATAGCCGAATACAC
Hsa mt-ND1 Reverse	GGGGGTTTAAGCTCCTAT
Mus mt-ND1 Forward	CCGAGCATCTTATCCACGCT
Mus mt-ND1 Reverse	ATGGTGGTACTCCCGCTGTA
Hsa CYGB Forward	CACAAGGTGGAACCGGTGTA
Hsa CYGB Reverse	TGGAGTTAGGGGTCCTACGG
Mus CYGB Forward	CCAGCCACTCTGCCCTCT
Mus CYGB Reverse	CCTCCTTTCGGGAAGTCGAG

Hsa, human; Mus, mice.

Supplementary Table 3. Effects of PACS-2 knockout on physical and biochemical values of mice.

	NC+WT	STZ+WT	NC+*Pacs-2*$^{-/-}$	STZ+*Pacs-2*$^{-/-}$
BW (g)	27.66±1.95	22.10±1.94*	26.14±1.66	20.64±2.03*
FBG (mmol/L)	7.78±1.23	26.44±2.69*	8.14±1.13	24.88±1.99*
KW/BW (mg/g)	5.17±0.32	7.46±0.42*	5.47±0.37	8.78±0.82*#
UAE (μg/24h)	19.62±4.39	181.11±34.02*	23.52±5.97	286.12±40.67*#
ACR(μg/mg)	23.19±6.32	223.80±53.13*	27.92±5.40	323.02±55.96*#
NAG (mg/L)	22.14±4.80	55.26±9.10*	19.60±3.19	76.06±11.19*#
Scr (μmol/L)	25.28±2.86	44.58±7.20*	29.70±4.68	61.16±10.87*#
BUN (mmol/L)	5.07±1.09	10.88±1.97*	6.12±1.25	15.23±2.41*#
ALT (U/L)	40.58±5.28	42.16±4.65	39.14±3.24	38.08±3.93
AST (U/L)	85.90±9.82	88.46±12.97	82.52±12.02	93.98±13.83
TG (mmol/L)	1.61±0.11	1.53±0.16	1.43±0.10	1.49±0.19
TC (mmol/L)	2.49±0.26	2.75±0.19	2.68±0.30	2.55±0.34

ACR, urinary albumin to creatinine ratio; ALT, alanine aminotransferase; AST, aspartate aminotransferase; BUN, blood urea nitrogen; BW, body weight; FBG, fasting blood glucose; KW, kidney weight; NAG, N-acetyl-β-D-glucosaminidase; Scr, serum creatinine; TC, total cholesterol; TG, triglyceride; UAE, urinary albumin excretion. n = 5. ANOVA followed by a post hoc Turkey's test was used for statistical analysis. All data are expressed as mean ± SD. * represents significant differences between STZ+WT and NC+WT or between STZ+*Pacs-2*$^{-/-}$ and NC+*Pacs-2*$^{-/-}$. # represents significant differences between STZ+*Pacs-2*$^{-/-}$ and STZ+WT.

Supplementary Table 4. Effects of PACS-2 overexpression on physical and biochemical values of mice.

	NC+ Ad-Con	STZ+ Ad-Con	NC+ Ad-PACS-2	STZ+ Ad-PACS-2
BW (g)	25.86±1.02	21.78±0.72*	25.48±1.19	20.7±0.97*
FBG (mmol/L)	8.74±1.13	28.06±4.27*	8.24±1.50	26.62±1.38*
KW/BW (mg/g)	5.58±0.43	8.31±0.84*	5.96±0.56	7.12±0.58*#
UAE (μg/24h)	14.43±4.61	149.8±28.91*	12.22±3.38	90.06±20.62*#
ACR(μg/mg)	17.83±4.01	187.3±39.76*	16.10±2.38	101.66±13.32*#
NAG (mg/L)	26.81±5.68	61.23±9.49*	25.76±4.35	44.15±8.97*#
Scr (μmol/L)	29.68±3.22	47.54±5.84*	27.38±5.33	37.76±5.16*#
BUN (mmol/L)	6.23±0.96	11.50±1.31*	5.89±1.22	7.09±0.91*#
ALT (U/L)	42.88±8.24	40.54±6.76	46.16±6.49	39.54±6.68
AST (U/L)	94.84±10.39	90.76±26.18	83.74±14.09	85.04±15.46
TG (mmol/L)	1.47±0.20	1.45±0.18	1.36±0.20	1.52±0.26
TC (mmol/L)	2.34±0.11	2.40±0.39	2.35±0.25	2.45±0.30

ACR, urinary albumin to creatinine ratio; ALT, alanine aminotransferase; AST, aspartate aminotransferase; BUN, blood urea nitrogen; BW, body weight; FBG, fasting blood glucose; KW, kidney weight; NAG, N-acetyl-β-D-glucosaminidase; Scr, serum creatinine; TC, total cholesterol; TG, triglyceride; UAE, urinary albumin excretion. n = 5. ANOVA followed by a post hoc Turkey's test was used for statistical analysis. All data are expressed as mean ± SD. * represents significant differences between STZ+Ad-Con and NC+Ad-Con or between STZ+Ad-PACS-2 and NC+Ad-PACS-2. # represents significant differences between STZ+Ad-PACS-2 and STZ+Ad-Con.

Supplementary Figures

Supplementary Figure 1

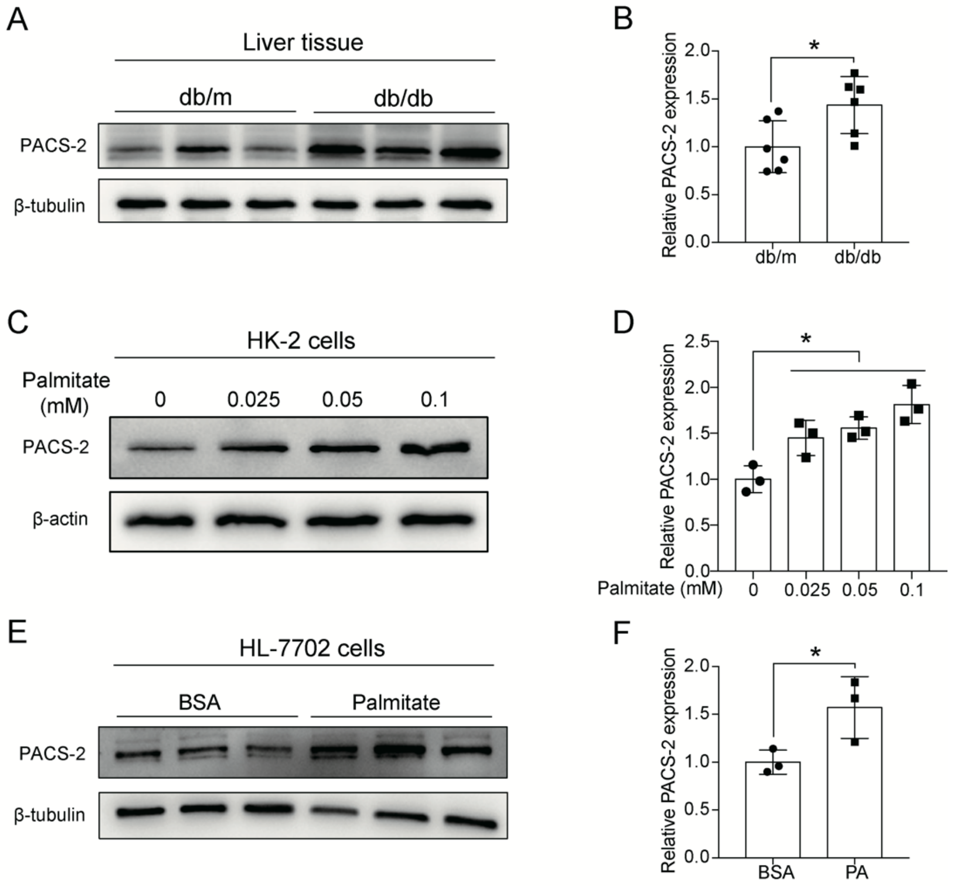

Supplementary Fig. 1: PACS-2 protein expression analyzed by Western blot analysis. (A-B) The protein expression and quantitative analysis of PACS-2 in the liver of db/m and db/db mice. (C-E) The protein expression and quantitative analysis of PACS-2 in HK-2 cells (C-D) or HL-7702 cells (E-F) stimulated by palmitate. n = 3. n = 6. All data are expressed as mean ± SD. * represents *P*<0.05.

Supplementary Figure 2

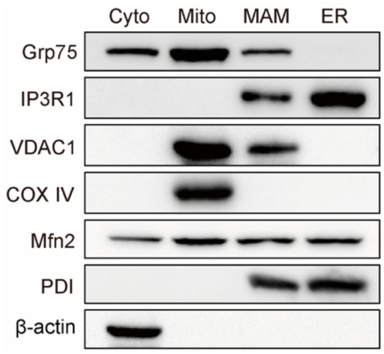

Supplementary Fig. 2: Validation of subcellular fractions isolated from mice kidneys by Western blot analysis.

Supplementary Figure 3

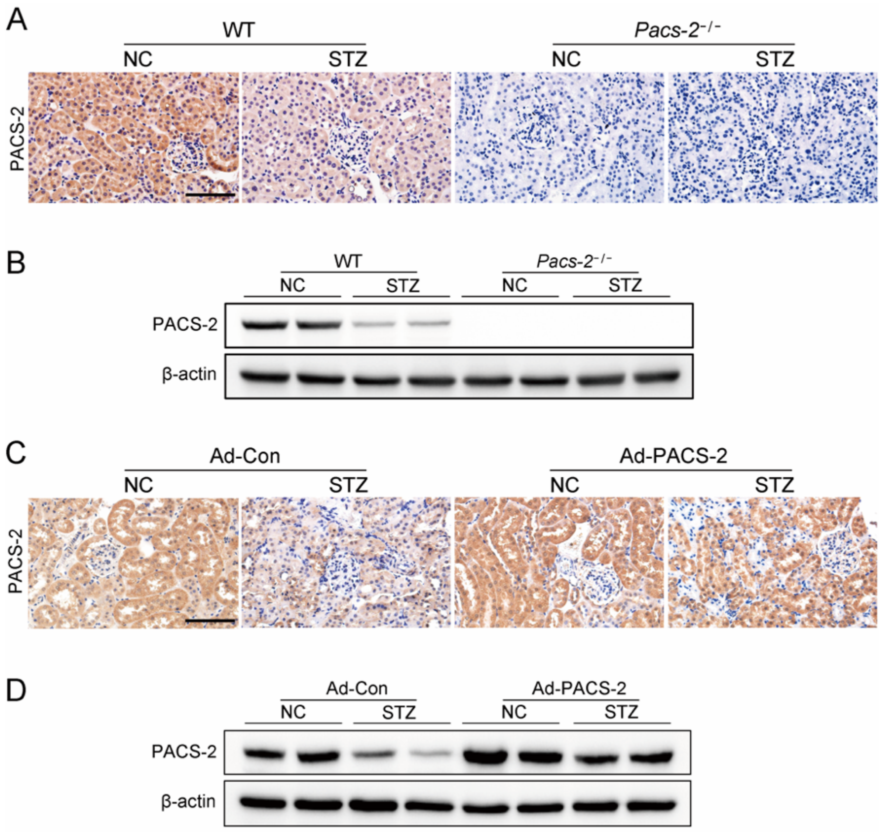

Supplementary Fig. 3: Renal PACS-2 expression levels in different mouse models. (A) and (C) Validation of PACS-2 knockout or overexpression by immunohistochemical analysis. Scale bar = 100μm. (B) and (D) Validation of PACS-2 knockout or overexpression by Western blot analysis.

Supplementary Figure 4

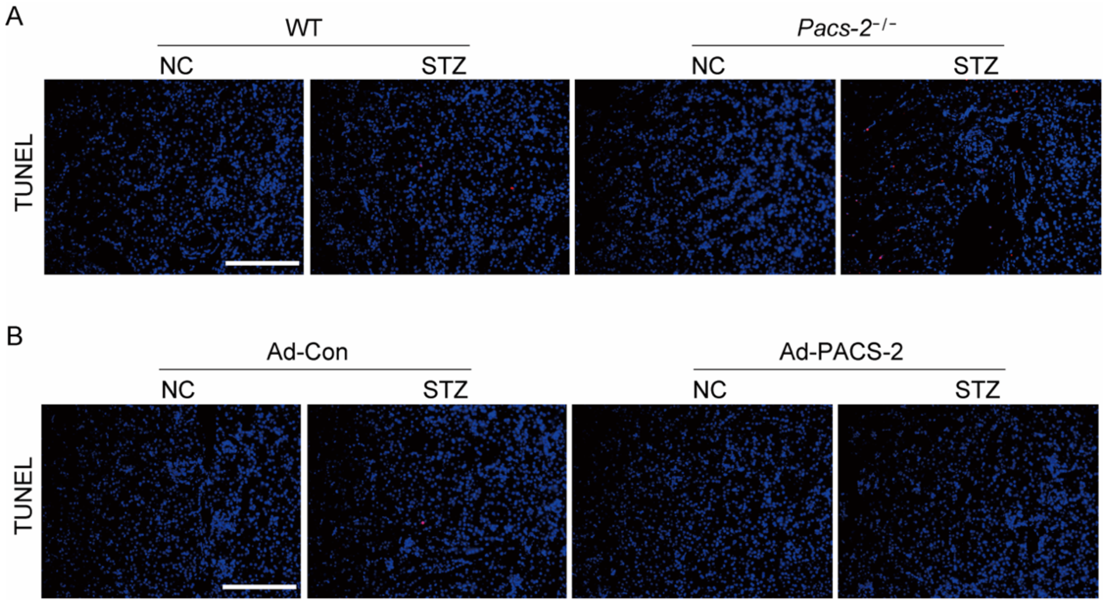

Supplementary Fig. 4: Representative images of TUNEL staining of kidney sections in PACS-2 knockout (A) or overexpression (B) mice. Scale bars = 100μm.

Supplementary Figure 5

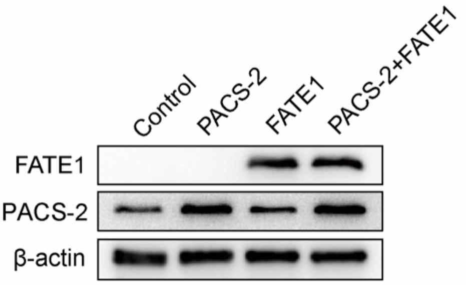

Supplementary Fig. 5: Validation of FATE1 or PACS-2 overexpression by Western blot analysis in HK-2 cell.
